# Charcot-Marie-Tooth disease: A case report initially manifested by bilateral vocal cord paralysis

**DOI:** 10.1016/j.rmcr.2024.102154

**Published:** 2025-01-08

**Authors:** Seyed Hossein Mirlohi, Sanaz Tajfirooz, Mitra Rouhi

**Affiliations:** a Pediatric Respiratory and Sleep Medicine Research Center, Children's Medical Center, Tehran University of Medical Sciences, Tehran, Iran; b Children's Medical Center, Pediatric Center of Excellence, Tehran, Iran

## Abstract

Charcot-Marie-Tooth is an inherited disorder involving multiple genes, causing progressive nerve damage affecting sensation and movement. The complexity of the condition often leads to various possible diagnoses along with neuropathic diseases, sometimes resulting in significant delays in diagnosis and treatment. Thorough clinical examinations, suspicion based on symptoms, electromyography, nerve conduction tests, and specific genomic testing can expedite diagnosis. Here, we will introduce a case of Charcot-Marie-Tooth disorder, initially presenting with stridor and hoarseness due to vocal cord paralysis, later progressing to atrophy and deformity of the limbs. Diagnosis was confirmed through whole genome sequencing, revealing mutations in genes associated with the disorder.

## 1 Introduction

Charcot-Marie-Tooth (CMT) disease is a rare and diverse set of genetic disorders impacting sensory and motor nerves, affecting about 1 in 2500 people worldwide [1,2]. Over the past decade, extensive research has focused on uncovering the genetic roots of CMT, revealing numerous mutations and genomic variations as primary contributors [3,4]. These genetic mechanisms, spanning from point mutations to copy number variations contribute to a wide range of symptoms and a complex genetic pattern [5]. However, diagnosing CMT is challenging due to its similarities with other acquired nerve disorders, often leading to delays in identification and treatment. Here, we will present a case of CMT, which was missed during initial clinical assessments, later confirmed through genetic testing.

## 2 Case presentation

An 8-year-old boy had been admitted to our medical center complaining of stridor and hoarseness. He was the second child of the family, and there were no similar complaints from any of his family members. His medical history included being hospitalized at age the age of 2.5 due to noisy breathing and was diagnosed with laryngomalacia. Later, due to his continuous symptoms, bronchoscopy was performed and he was diagnosed as bilateral vocal cord paralysis at Tehran Children's Medical Center and then he underwent tracheostomy. However, Laser arytenoidectomy was done on him afterward. At age 7, he started showing symptoms of peripheral neuromyopathy, prompting further investigations. Electromyography (EMG) and nerve conduction studies (NCS) showed reduced sensory nerve action potentials (SNAPs) and compound muscle action potentials (CMAP) and H-reflexes, raising suspicion of CMT. Confirmatory testing through whole-exome sequencing and Sanger sequencing revealed mutations in several genes associated with CMT. Based on his genetic assessment results, some genomic variants had been discovered including the disrupted COG6 gene variant (c.1584+5dup), as well as several single neucleotide polymorphisms on the genes of MFN2 (842G > C), PKLR (1456C > G), PAH (c289C > T), SEPSECS (C.478G > A), MCCC2 (c.1236C > T), PLOD1 (c.1991C > T), MMUT (c.424G > A), and ETFDH (c.524G > A) confirming the definitive diagnosis of CMT. In the evaluation of genetic variants in his family members, both of his parents were carriers for PKLR gene polymorphism (1456C > G) and his mother was a carrier for MFN2 gene polymorphism (842G > C).

## 3 Discussion

Since the discovery of CMT in 1886, extensive biological and genomic studies have been conducted to understand the pathophysiology of CMT in order to develop early diagnostic techniques [6]. Clinically, CMT is marked by both motor and sensory deficits, indicating a sensory-motor neuropathy disorder, with symptoms like muscle atrophy, limb weakness, and diminished deep tendon reflexes [7]. In chronic conditions, motor neuropathy may lead to deformities of the limbs [8]. In certain cases, sensory deficits may occur as decreased pain and temperature sensation [9]. Electromyography (EMG) and nerve conduction studies (NCS) results can suggest that genetic tests are necessary for the patient [10]. Nevertheless, EMG and NCS are also essential in classifying different types of peripheral neuropathies [11]. Moreover, electrophysiological tests, such as EMG, NCS and genetic testing are vital in order to distinguish between demyelinating and axonal forms of CMT [12]. Mendelian hereditary forms of the disease may also be appeared for CMT [13]. Thus, genetic testing has transformed diagnostic methods, allowing for the detection of various mutations associated with CMT. Furthermore, many genes related to synthesizing structural proteins involved in myelination include (PMP22, MPZ), proteins of axonal transport (NEFL), genes involved in Schwann cell differentiation (EGR2), gene controlling members of signal transduction pathways (NDGR1) and genes regulating mitochondrial function (MFN2) have been identified to be responsible for the appearance and progression of CMT [14,15].

## 4 Conclusion

This case is distinctive as the patient was admitted based on non-specific symptoms such as stridor and hoarseness and CMT had not been considered initially which led to delay in the right diagnosis. Based on the clinical manifestations, by taking the right approach like neurological and muscular evaluations followed by genetic tests, the final diagnosis was CMT. This case aims to highlight the importance of considering CMT in patients with bilateral vocal cord paralysis and emphasizes the necessity of neurological and genetic assessments.

## CRediT authorship contribution statement

**Seyed Hossein Mirlohi:** Conceptualization, Data curation, Supervision, Writing – review & editing. **Sanaz Tajfirooz:** Conceptualization, Data curation, Investigation, Supervision, Writing – original draft, Writing – review & editing. **Mitra Rouhi:** Conceptualization, Data curation, Supervision.

## Declarations

Ethics approval and consent to participate the Ethics Committee of Tehran University of Medical Sciences approved this study. (Approval ID: **IR.TUMS.CHMC.REC.1403.102**).

We extend our gratitude to the patient and his parents for generously contributing their time and valuable information to the study.

We declare that the authors have no conflict of interest.

## Declaration of competing interest

The authors declare that they have no known competing financial interests or personal relationships that could have appeared to influence the work reported in this article.
